# Osteosarcoma-Derived Small Extracellular Vesicles Enhance Tumor Metastasis and Suppress Osteoclastogenesis by miR-146a-5p

**DOI:** 10.3389/fonc.2021.667109

**Published:** 2021-05-04

**Authors:** Yoshihiro Araki, Hisaki Aiba, Takeshi Yoshida, Norio Yamamoto, Katsuhiro Hayashi, Akihiko Takeuchi, Shinji Miwa, Kentaro Igarashi, Tuan D. Nguyen, Kiyo-aki Ishii, Takayuki Nojima, Satoru Takahashi, Hideki Murakami, Hiroyuki Tsuchiya, Rikinari Hanayama

**Affiliations:** ^1^ Department of Immunology, Kanazawa University Graduate School of Medical Sciences, Kanazawa, Japan; ^2^ Department of Orthopaedic Surgery, Kanazawa University Graduate School of Medical Sciences, Kanazawa, Japan; ^3^ Department of Orthopaedic Surgery, Nagoya City University Graduate School of Medical Sciences, Nagoya, Japan; ^4^ Department of Experimental Pathology and Tumor Biology, Nagoya City University Graduate School of Medical Sciences, Nagoya, Japan; ^5^ WPI Nano Life Science Institute (NanoLSI), Kanazawa University, Kanazawa, Japan; ^6^ Department of Integrative Medicine for Longevity, Kanazawa University Graduate School of Medical Sciences, Kanazawa, Japan; ^7^ Department of Pathology, Kanazawa University Graduate School of Medical Sciences, Kanazawa, Japan

**Keywords:** osteosarcoma, extracellular vesicle, osteoclast, prognosis, NF-κB signal pathway, microRNA

## Abstract

Osteosarcoma is the most frequent type of primary bone tumor in children and adolescents, thus care for patients with malignant osteosarcoma is strongly required. The roles of small extracellular vesicles (SEVs) in enhancing metastases have been demonstrated in multiple tumors, but they are still poorly understood in osteosarcoma. Hence, this study investigated the effects of SEVs on progression and the tumor microenvironment in mice and patients. In an orthotopic implantation study, we found that osteosarcoma-derived SEVs had the potential to enhance metastases and angiogenesis. In addition, osteosarcoma-derived SEVs decreased the number of mature osteoclasts *in vivo*. *In vitro* osteoclastogenesis studies revealed that the inhibition of osteoclast maturation by osteosarcoma-derived SEVs was mediated by suppressing the NF-κB signal pathway. MicroRNA analysis of SEVs from different malignant human osteosarcomas revealed that miR-146a-5p was involved in the inhibition of osteoclastogenesis. In osteosarcoma patients, lower numbers of osteoclasts in biopsy specimens at the first visits were correlated with higher malignancy. These findings indicated that osteosarcoma-derived SEVs enhance distant metastasis of osteosarcomas by inhibiting osteoclast maturation, which may be a useful prognostic marker. This diagnostic method may enable to predict malignancy at early stage, and help to provide optimal care to patients with risk of high malignancy.

## Introduction

Osteosarcoma (OS) is the most frequent type of primary bone tumor in children and adolescents (1). OS is treated by surgical tumor resection combined with neoadjuvant and adjuvant chemotherapy to eradicate micrometastases from the primary site. Even when no overt metastases are observed at first diagnosis, many patients have sub-clinical metastases (2). The 5-year survival rate of patients with metastases is only approximately 20%, in contrast to approximately 70–80% for patients without metastases (3). Preventing metastases has been suggested to have potential to prolong survival time (4). Accordingly, recent studies have focused on the tumor microenvironment to identify potential factors involved in invasion or metastases (5, 6).

Because preclinical studies had shown that zoledronic acid (ZA) has a direct antiproliferative effect on OS cell lines (7, 8), Phase III clinical trials were conducted in France (OS2006) and China to examine its antitumor effect. In contrast, the two clinical trials reported that ZA either lacked or had negative effects on OS patients (9, 10), suggesting that osteoclasts (OCs) play unknown roles in the progression of OS. Only one study reported that treatment with ZA reduces the number of OCs and increases metastasis risk (11). However, the cause of the association between OCs and metastatic risk remains unknown.

Recently, small extracellular vesicles (SEVs) have attracted attention as important players in tumor metastases. SEVs are small membrane vesicles consisting of a lipid bilayer incorporating proteins, mRNAs, and microRNAs (miRNAs) as cargos (12). SEVs, particularly exosomes, are generated as intraluminal vesicles in multivesicular endosomes, and knockdown of the endosomal sorting complex required for transport genes, including *TSG101*, is known to reduce the secretion of SEVs (13). It has been reported that tumor-derived SEVs can alter the microenvironment by promoting angiogenesis or metastasis in several tumors (14). The OS microenvironment is composed of different types of stromal cells, including OCs, osteoblasts, osteocytes, chondrocytes, mesenchymal stem cells, hematopoietic cells, endothelial cells, and various immunocytes, which play multiple roles in proliferation, angiogenesis, and metastasis (6, 15). OCs, bone tissue-resident macrophages, are considered most susceptible to OS-SEVs due to diverse SEV receptor expression on macrophages (16, 17). However, there are few reports regarding the functions of SEVs secreted from OS (OS-SEVs), except for one indicating that they are transferred to a lesion destined to metastasize before establishing metastasis (18).

In this study, we investigated the effects of OS-SEVs on the tumor microenvironment (i.e. OCs) and their relationship with tumor malignancy. To clarify the effects of OS-SEVs on OCs and tumor progression, OS cell lines with suppressed SEV secretion were generated *via* KO of the *Tsg101* gene. An orthotopic implantation murine OS study revealed that OS-SEVs have potential to facilitate distant metastases and angiogenesis and inhibit the osteoclastogenesis. Furthermore, we analyzed miRNAs contained in SEVs from different malignant human OS cell lines and identified miR-146a-5p as a potent inhibitor for osteoclastogenesis. Finally, a study in OS patients revealed that lower numbers of OCs are associated with shorter overall survival. These findings indicated that larger numbers of OS-SEVs with greater malignancy suppress the osteoclastogenesis and enhance distant metastasis.

## Materials and Methods

### Plasmids

The pX330Puro plasmid was constructed by inserting a puromycin-resistant gene cassette into the *Not*I site of the pX330-U6-Chimeric BB-CBh-hSpCas9 plasmid (Addgene, Watertown, MA, USA) (19). To knock out mouse *Tsg101* gene, we generated the pX330Puro-TSG101 plasmid targeting the CCTACTAGTTCAATGACTATTA sequence.

### Cells

The highly aggressive murine OS cell line LM8 was obtained from RIKEN BRC (Ibaraki, Japan). NIH/3T3 cells, HOS cells (non-aggressive, wild-type k-Ras, human OS), and 143B cells (highly aggressive; k-Ras activated, human OS) (20, 21) were obtained from the ATCC (Manassas, VA, USA). These cells were cultured in Advanced DMEM (Thermo Fisher Scientific, Waltham, MA, USA) with 2% heat-inactivated FBS (Bio-West, Nuaillé, France). LM8-WT or LM8 TSG101-KO cells were generated by the CRISPR–Cas9 system. The pX330Puro or pX330Puro-TSG101 plasmid was transfected into LM8 cells using ViaFect (Promega, Madison, WI, USA). LM8 TSG101-KO cells were monocloned, resulting in the generation of two monoclonal LM8 TSG101-KO cell lines (KO1 and KO2). LM8-WT cells were maintained as a polyclonal population.

### Orthotopic Implantation Murine OS Model

LM8-WT or LM8 TSG101-KO cells **(**1 × 10^5^ cells) were implanted in the distal femurs of 8-week-old, male C3H/He mice (Japan SLC, Tokyo, Japan) with Matrigel (Corning, Corning, NY, USA). When the implanted tumor reached 2,000 mm^3^ or at 4 weeks post-implantation (sham group), the lungs and femurs were collected. The tissues were embedded in paraffin, and were subjected to H&E staining. For immunohistochemistry (IHC), deparaffinized sections were incubated with antibodies against cathepsin K (Abcam, Cambridge, United Kingdom, Cat# ab19027), Ki67 (Abcam, Cat# ab15580), CD31 (Abcam, Cat# ab28364), or vascular endothelial growth factor (VEGF; Santa Cruz Biotechnology, Santa Cruz, CA, USA, Cat# sc-7269), followed by treatment with an HRP-conjugated secondary antibody and DAB-Chromogen staining. The number of Ki67-positive cells or area of VEGF-positive cells was counted by microscopy BZ-X710 and BZ-X Analyzer (KEYENCE, Osaka, Japan). This study was approved by the Institutional Animal Care of Kanazawa University.

### Isolation of SEVs

To isolate SEVs from LM8, NIH/3T3, 143B or HOS cells, the cells were cultured in Advanced DMEM with 2% SEV-depleted FBS prepared using a previously described method (22). At about 80% confluency, the conditioned medium was harvested, and sequentially centrifuged at 300 × *g* for 5 min, 2,000 × *g* for 20 min, and 10,000 × *g* for 30 min. Following a 2-h ultra-centrifugation at 100,000 × *g* using a S50ST rotor (Hitachi, Tokyo, Japan), SEVs were collected from the pellet, washed with PBS (–) by repeated ultra-centrifugation (23), and finally reconstituted in PBS(-). Size distribution and quantity of the SEVs were determined by nanoparticle tracking analysis using NanoSIGHT™ LM10 (Malvern Panalytical, Malvern, United Kingdom) (**Supplementary Figure 1**). Characterization of isolated SEVs was performed following MISEV2018 guidelines (24). To prepare PKH-labeled LM8-SEVs, the isolated SEV pellet was stained with 1 µL of PKH26 (Merck, Darmstadt, Germany) for 3 min, followed by quenching the staining reaction by adding 1% BSA/PBS(-). Excess staining reagents were removed by 2 h of ultra-centrifugation, and the pellet of PKH-labeled SEVs was reconstituted in PBS(-).

### Osteoclastogenesis

Bone marrow was collected from femurs and tibias of 8-week-old, male C3H/He mice (Japan SLC) and cultured for 3 days at a density of 5 × 10^6^ cells/100-mm dish in MEMα (FUJIFILM Wako, Osaka, Japan) supplemented with 10% FBS and 100 ng/mL of macrophage colony-stimulating factor (M-CSF; BioLegend, San Diego, CA, USA). To induce osteoclast precursor cells (OPCs), the cells were cultured for additional 3 days at 5 × 10^5^ cells/100-mm dish. For osteoclastogenesis, OPCs were cultured for 5 days at 1 × 10^5^ cells/12-well plate in MEMα with 10% FBS, 20 ng/mL M-CSF, and 50 ng/mL of receptor activator of NF-kappaB ligand (RANKL; Biolegend). To determine effects of SEVs, 1 × 10^10^ particles/mL or indicated doses of SEVs were added to the medium at the beginning of osteoclastogenesis. To determine the effects of miRNA, 60 pmol of miRNA mimic transfection control with Dy547 (miR-Ctrl) or hsa-miR-146a-5p mimic (Horizon Discovery, Cambridgeshire, United Kingdom) was transfected into OPCs using Lipofectamine RNAiMAX (Thermo Fisher Scientific) at the beginning of osteoclastogenesis. Cellular growth was evaluated using WST-8 assay reagent (Nacalai Tesque, Kyoto, Japan). TRAP staining was performed after 5 days of osteoclastogenesis by using an Acid Phosphatase Leukocyte Kit (Merck), and was evaluated using microscopy BZ-X710 and BZ-X Analyzer.

### Real-Time PCR

To detect mRNA, total RNA extraction was performed using a High Pure RNA Isolation Kit (Roche, Basel, Switzerland). The cDNA was reverse-transcribed using 100 ng RNA and 10 µL reaction mix from ReverTra Ace qPCR RT Kit (TOYOBO, Osaka, Japan). After diluting the cDNA with 90 µL of dH2O, 4 µL of cDNA was reacted with 16 µL of the Fast SYBR Green Master Mix (Thermo Fisher Scientific) in a LightCycler96 system (Roche). At the end of the real-time PCR, specificity was confirmed with the help of melting peak analysis. Paired primers are listed in **Supplementary Table 1**. mRNA expression of each gene was normalized to *Gapdh*. To detect miRNA, total small RNA extraction was performed using a FastGene RNA Basic Kit and FastGene miRNA Enhancer (NIPPON Genetics, Tokyo, Japan). The cDNA was reverse-transcribed using 250 ng of the total small RNA and 10 µL reaction mix from Mir-X miRNA First-Strand Synthesis (Takara Bio, Shiga, Japan). After diluting the cDNA with 90 µL of dH2O, 2 µL of cDNA was reacted with 23 µL of TB Green Advantage reaction mix in a LightCycler96 system. At the end of the real-time PCR, specificity was confirmed by melting peak analysis. Paired primers are listed in **Supplementary Table 1**. The expression of hsa-miR-146a-5p was normalized to that of U6.

### Western Blot Analysis

In NF-κB pathway analysis, OPCs were seeded at 1 × 10^5^ cells/12-well plate and pre-cultured for 12 h in MEMα with 10% FBS, 20 ng/mL M-CSF, and 1 × 10^10^ particles/mL of SEVs. The medium was changed to MEMα with 10% FBS, 20 ng/mL M-CSF, and 50 ng/mL of RANKL. After RANKL-stimulation, cells were washed with PBS(-) and lysed in RIPA buffer with protease inhibitor (FUJIFILM Wako) and phosphatase inhibitor (Nacalai Tesque). For the analysis of TRAF6 expression, 1 × 10^5^ cells of OPCs were treated with 1 × 10^10^ particles/mL of SEVs or 60 pmol of miRNA mimic in MEMα with 10% FBS and 20 ng/mL M-CSF. After 48 h of incubation, cells were washed with PBS(-) and lysed in RIPA buffer with protease inhibitor. The lysates were subjected to western blot analysis. Primary antibodies for IκBα, phospho-IκBα, NF-κB, and phospho-NF-κB were purchased from Cell Signaling Technology (Cat# 4818, 2859, 8242, and 3033, respectively), and those for GAPDH and TRAF6, from MBL (Woburn, MA, USA, Cat# M171-38) and BioLegend (Cat# T2-1SC), respectively. HRP-conjugated anti-rabbit IgG and anti-mouse IgG were purchased from Cell Signaling Technology (Cat# 7076) and Jackson ImmunoResearch (Cat# 715-035-151), respectively. The proteins were detected using SuperSignal West Pico Chemiluminescent Substrate (Thermo Fisher Scientific) and ImageQuant LAS4000 Mini (GE Healthcare, Chicago, IL, USA). Intensities of the bands were determined by ImageJ software (NIH, Bethesda, MD, USA). The intensity of each band was normalized to GAPDH.

### SEV-ELISA

Cells were seeded at 5 × 10^4^ cells/24-well plate in Advanced DMEM with 2% SEV-depleted FBS. After 24 h of culture, the culture supernatants were collected and sequentially centrifuged at 300 × *g* for 5 min, 2,000 × *g* for 20 min, and 10,000 × *g* for 30 min. The resulting supernatants were subjected to ELISA, following a similar method described previously (22). SEVs from human cell lines were detected using antibodies against human CD9, CD63, or CD81 (all from BioLegend, Cat# 312102, 353014, or 349501) and an HRP-conjugated anti-mouse IgG (Jackson ImmunoResearch, West Grove, PA, USA, Cat# 715-035-151).

### Microarray Analysis of miRNA in SEVs

SEVs were isolated from conditioned medium of HOS cells or 143B cells using MagCapture Exosome Isolation Kit (FUJIFILM Wako). MiRNAs contained in the SEVs were detected using Human miRNA Oligo chip (Toray Industries Inc., Tokyo, Japan). The value of each miRNA was normalized by correcting the median of the signal intensities to 25, resulting in a global normalization value. Each ratio of miRNA in 143B-SEV to HOS-SEV was calculated by dividing the global normalization value of 143B-SEV by that of HOS-SEV.

### Clinical Samples

Biopsy specimens from all the chemotherapy-naïve primary OS patients were collected from the pathological database between 1999 and 2018. Patients with low-grade OS, dedifferentiated OS, multicentric OS, and secondary OS were excluded. Those who had undergone biopsy or any treatment at other hospitals and those with less than 3 months follow-up after diagnosis were also excluded. Distant metastases were determined by multimodal radiological examinations, including computed tomography, bone scintigraphy, and PET, in all patients at first visit. The presence of OCs was determined by IHC using biopsy specimens around the osteoid produced by tumors using an antibody against cathepsin K (Santa Cruz Biotechnology, Cat# sc-48353). OCs were defined as cathepsin K-positive cells >1,000 μm^2^ in area. Based on the analysis of three IHC images, patients were categorized into the OC(+) group (≥ 5 OCs/field in at least two images) or OC(−) group (< 5 OCs/field in at least two images).

### Ethical Approval

This retrospective study of patient specimens was approved by the ethical committee of Kanazawa University Hospital (Clinical Trial Number 2310 and 3249) in compliance with the guidelines of the Helsinki Declaration revised in 2013.

### Statistical Analysis

To analyze patients’ oncological outcomes, Kaplan–Meier curves were constructed, and differences between each group were compared using the log-rank test. Metastasis-free survival, recurrence-free survival, progression-free survival, and overall survival was defined as the time from definitive diagnosis to distant metastasis or death, from definitive diagnosis to recurrence or death, from definitive diagnosis to distant metastasis, recurrence, lesion increases, or death, and from definitive diagnosis to death, respectively. The chi-squared test was used for statistical analysis of categorical data. For numerical data, Student’s *t*-tests or Mann–Whitney U tests (two-sided) were used, as appropriate, based on Shapiro–Wilk analysis of the distribution. These statistical analyses were performed using IBM SPSS, version 24 (IBM). The analyses for diagnostic accuracy were performed using EZR software (Jichi Medical University) (25).

## Results

### OS Cells With Impaired SEV Secretion Show Low Malignancy in Orthotopic Implantation Model

**Figure 1:**
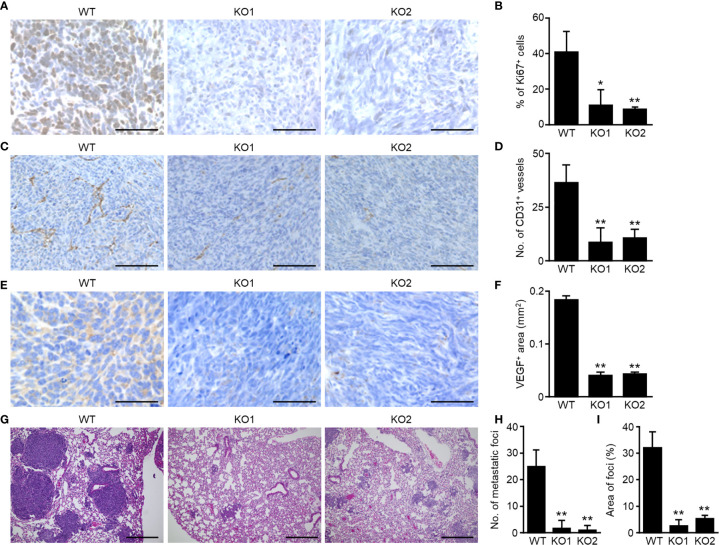
Decreased malignancy of SEV-suppressed OSs in orthotopic implantation murine model. Murine OS LM8-WT, TSG101-KO1, or TSG101-KO2 cells were implanted in distal femurs of C3H/He mice. When the tumors grew to 2,000 mm^3^, the femurs and lungs were collected from mice. The femurs were subjected to immunohistochemical staining of Ki67 **(A)**, CD31 **(C)**, or VEGF **(E)**. The percentage of Ki67-positive cells per total cells **(B)**, number of CD31-positive vessels per mm^2^ **(D)**, or area of VEGF-positive regions per mm^2^ **(F)** was measured. **(G)** The lungs were subjected to H&E staining. Number **(H)** or area **(I)** of metastatic foci was calculated. Magnifications of images are ×40 in **(A**, **E)**, ×20 in **(C)**, and ×10 in **(G)** Scale bars indicate 50 µm in **(A**, **E)**, 100 µm in **(C)**, and 300 µm in **(G)** Values in **(B**, **D**, **F**, **H**, **I)** represent the mean ± s.d. *P < 0.05, **P < 0.01 versus WT (Student’s *t*-test).

To analyze the effects of SEVs in OS, we generated mouse OS LM8 cells with impaired SEV secretion by knocking out the *Tsg101* gene (**Supplementary Figure 2A**). Two independent clones of LM8 TSG101-KO cells (KO1 and KO2) showed approximately 40% SEV reduction (**Supplementary Figures 2B, C** **)** but no difference in cell proliferation *in vitro* (**Supplementary Figure 2D**). To determine the function of OS-SEVs in OS progression *in vivo*, LM8 TSG101-KO cells were embedded into distal femurs of mice. Contrary to the *in vitro* result, growth of the LM8 TSG101-KO cells was slower than that of LM8 WT cells *in vivo* (**Supplementary Figure 2E**). To eliminate potential bias, all examinations were performed when tumors grew to identical sizes. Ki67 staining in primary lesions showed 75% reduced expression in LM8 TSG101-KO cells (**Figures 1A, B** **)**. Considering that there was no difference in cell proliferation *in vitro*, LM8-SEVs might have the potential to create a favorable microenvironment for tumor growth by influencing neighboring cells *in vivo*. Additionally, angiogenesis was dramatically reduced by LM8-SEV suppression, as indicated by a 75% reduction in CD31-positive vessels (**Figures 1C, D** **)** and an approximate 75% reduction in VEGF-positive areas (**Figures 1E, F** **)** in mice bearing TSG101-KO cells, possibly contributing to the slow tumor growth of TSG101-KO cells. In evaluations of OS-SEV effects on distant metastasis based on lung specimens, mice bearing TSG101-KO cells had > 15-fold fewer metastatic foci and 6-fold smaller areas of metastatic lesions than those with WT cells, when the primary lesion of each tumor grew to identical sizes (**Figures 1G–I** **)**. These data indicated that OS-SEVs altered the tumor microenvironment to be more favorable for tumors, enhancing tumor growth, angiogenesis, and distant metastasis.

### OS-SEVs Inhibit Osteoclastogenesis

**Figure 2:**
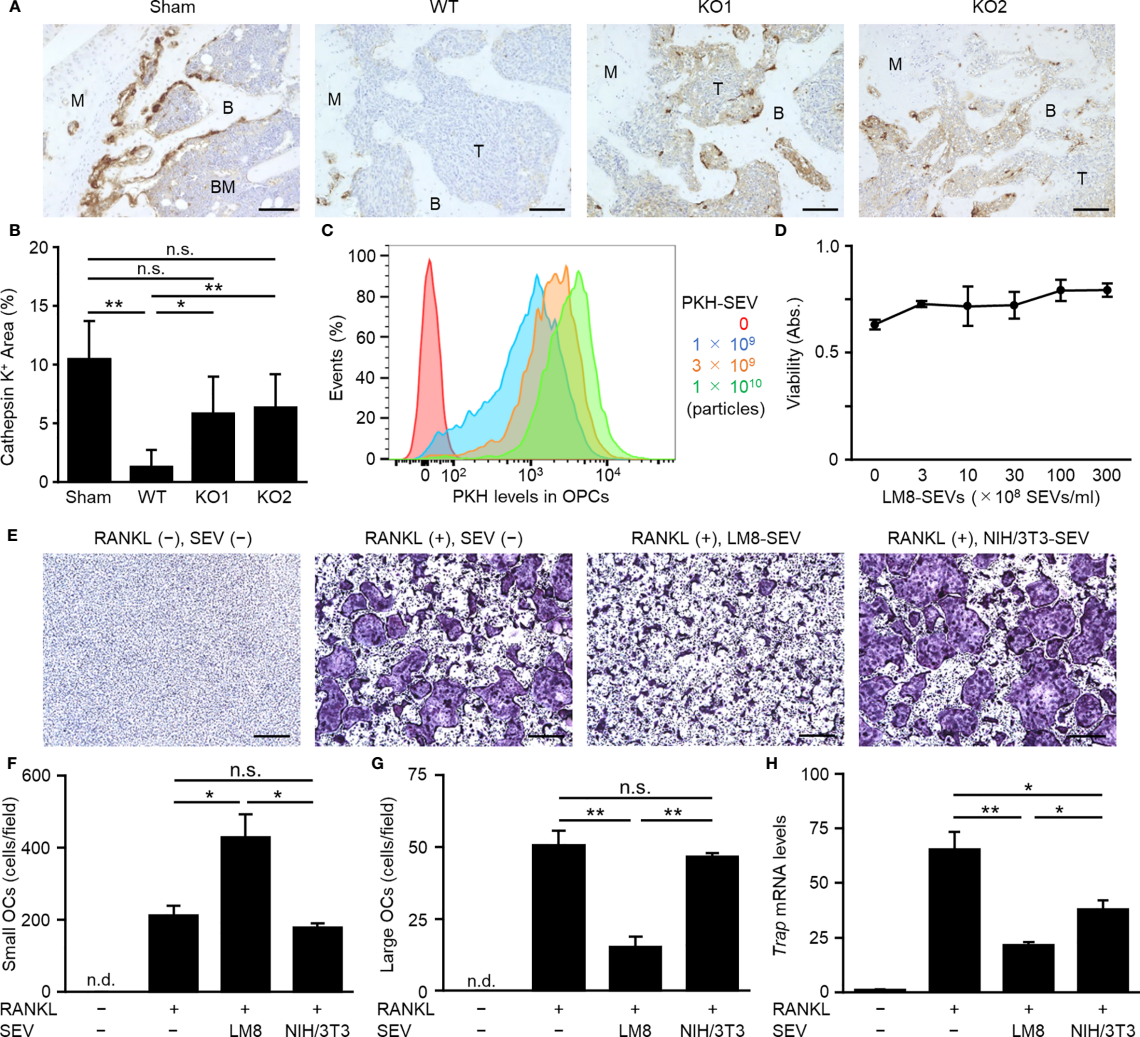
Suppression of osteoclastogenesis by OS-SEVs. **(A)** LM8-WT, TSG101-KO1, or TSG101-KO2 cells were implanted in distal femurs of C3H/He mice. When the tumors grew to 2,000 mm^3^, the femurs were subjected to immunohistochemical staining for cathepsin K. Images were taken at ×20 magnification. B, BM, M, or T in the images indicate bone, bone marrow, metaphysis or tumor, respectively. Scale bars show 100 µm. **(B)** The area of cathepsin K-positive cells in a field was calculated. Values represent the mean ± s.d. *P < 0.05, **P < 0.01, n.s. not significant versus sham or WT (Student’s *t*-test with Bonferroni correction). **(C)** OPCs (1 × 10^5^ cells) were cultured in media containing each dose of PKH-labeled LM8-SEVs for 24 h, and were analyzed by a flow cytometer. **(D)** OPCs were cultured for 2 days in media containing each dose of LM8-SEVs, and cellular viabilities of the OPCs were measured by a WST-8 assay. **(E)** OPCs were cultured for 5 days in media with or without each SEVs and with or without RANKL. Images of TRAP-staining were taken at ×4 magnification. Scale bars represent 500 µm. **(F, G)** The area of TRAP-positive cells shown in **(E)** was measured. Numbers of small OCs with a 1,000–10,000 μm^2^ TRAP-positive area or large OCs with ≥ 10,000 μm^2^ per field were calculated. **(H)** Relative expression of *Trap* and *Gapdh* genes in the OPCs cultured for 5 days with or without RANKL or SEVs was detected by real-time PCR. The expression level of the *Trap* gene was normalized to *Gapdh* expression and represented as a relative value to the RANKL(−), SEV(−) group. Values in **(D)**, **(F–H)** represent the mean ± s.d. *P < 0.05, **P < 0.01 versus the RANKL(+), SEV(−) group (Student’s *t*-test with Bonferroni correction). n.d., not detected.

OCs are known as one of the important cells constituting the tumor microenvironment in OS (26). IHC of cathepsin K showed that OCs in mice bearing LM8-WT cells were drastically decreased compared to those in sham mice, whereas impaired secretion of OS-SEVs by TSG101-KO abrogated the decrease in OCs (**Figures 2A, B** **)**. Transfer analysis *in vitro* using PKH-labeled SEVs showed the efficient transfer of LM8-SEVs into OPCs in a dose-dependent manner (**Figure 2C**). A WST assay revealed that LM8-SEVs did not show cytotoxicity towards OPCs at any dose (**Figure 2D**). However, LM8-SEV treatment decreased the number of large OCs (> 10,000 µm^2^) containing over 10 nuclei and increased the number of small OCs (1,000–10,000 µm^2^) containing approximately 3–9 nuclei (**Figures 2E–G**). Furthermore, LM8-SEVs suppressed *Trap* mRNA expression by approximately 70% compared to that in mature OCs (**Figure 2H**). As these suppressive effects were not seen in the control NIH/3T3-SEV-treated group, molecules contained specifically in LM8-SEVs might be responsible. These results indicated that OS-SEVs strongly inhibited osteoclastogenesis and increased the number of small immature OCs, raising the possibility that decreases in mature OCs might be caused by the osteoclastogenesis inhibition by OS-SEVs.

### OS-SEVs Inhibit Osteoclastogenesis by Suppressing the NF-κB Pathway

**Figure 3:**
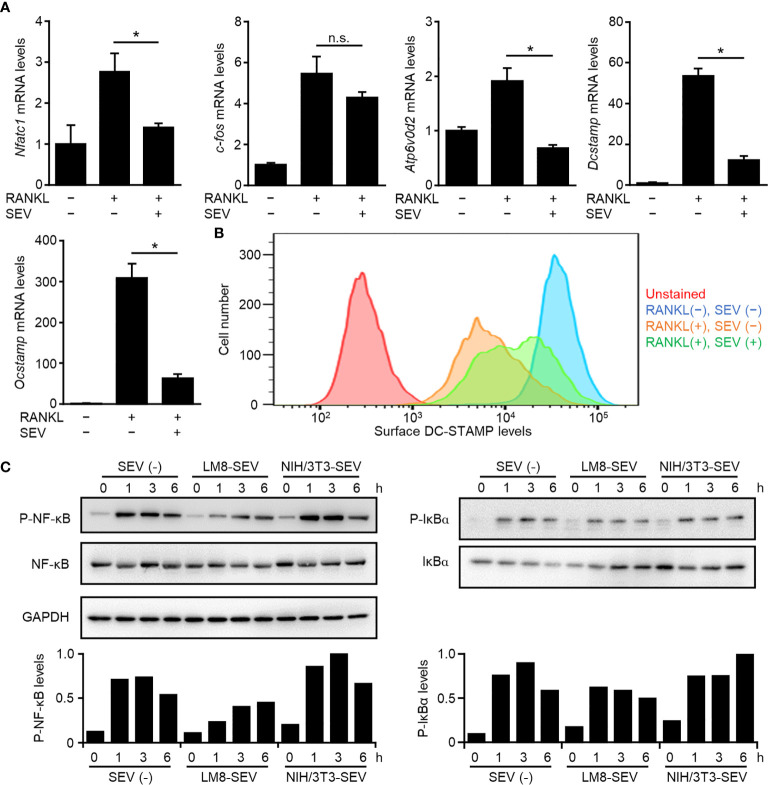
Suppression of genes involved in osteoclastogenesis and NF-κB pathway by LM8-SEVs. **(A)** OPCs were stimulated with or without RANKL or LM8-SEVs for 48 h. Expression of *Nfatc1*, *c-fos*, *Atp6v0d2*, *Dcstamp*, *Ocstamp*, and *Gapdh* genes was detected by real-time PCR. The expression level of each gene was normalized to *Gapdh* expression and represented as a relative value to the RANKL(−), SEV(−) group. Values represent the mean ± s.d. *P < 0.01, n.s., not significant versus the RANKL(+), SEV(−) group (Student’s *t*-test). **(B)** After a 48-h stimulation with or without RANKL and LM8-SEVs, surface DC-STAMP were stained with the anti-DC-STAMP antibody (1A2; Merck), and were analyzed with a flow cytometer. **(C)** OPCs were pre-cultured in media without SEVs or with each SEVs for 12 h, and stimulated with RANKL for the indicated time. Pphospho-NF-κB, phospho-IκBα, or GAPDH were detected in western blot. The band intensities were normalized to GAPDH. Relative values to the highest sample are shown in the bar graphs.

To determine the mechanism underlying the suppression of osteoclastogenesis by OS-SEVs, we analyzed gene expression of two key transcription factors (*c-fos* and *Nfatc1*) and three key molecules (*Dcstamp*, *Atp6v0d2*, and *Ocstamp*) involved in osteoclastogenesis (27, 28). Transfer of LM8-SEVs into OPCs had no effect on the expression of *c-fos* mRNA but strongly suppressed the expression of *Nfatc1* mRNA (**Figure 3A**). The suppression of *Nfatc1* might inhibit osteoclastogenesis, because NFATc1 is known to induce several key fusion mediators, including *Dcstamp*, *Atp6v0d2*, and *Ocstamp (* 28). As expected, real-time PCR analysis showed decreased mRNA expression levels of these markers (**Figure 3A**). Recently, multiple researchers have reported that DC-STAMP transport to the endoplasmic reticulum (ER) are important in osteoclastogenesis (20, 27). Consistently, our flow cytometric analysis showed that RANKL-stimulation induced the disappearance of DC-STAMP from the cellular surface, whereas LM8-SEVs suppressed this (**Figure 3B**). These findings indicated that OS-SEVs suppressed the expression of important genes involved in osteoclastogenesis and disturbed the ER transfer of DC-STAMP. The key transcription factor NFATc1 is known to be induced mainly by NF-κB or the MAPK pathway in RANKL-dependent osteoclastogenesis (28). We investigated the effects of OS-SEVs on the MAPK pathways and found small effects (**Supplementary Figure 3**). Conversely, the phosphorylation of IκBα or NF-κB in LM8-SEV-treated OPCs was lower than that in untreated or NIH/3T3-SEV-treated OPCs 1–6 h after RANKL stimulation (**Figure 3C**). These results suggested that OS-SEVs inhibited osteoclastogenesis by affecting proteins upstream of IκBα in the NF-κB pathway.

### MiR-146a-5p Contained in OS-SEVs Inhibits Osteoclastogenesis

**Figure 4:**
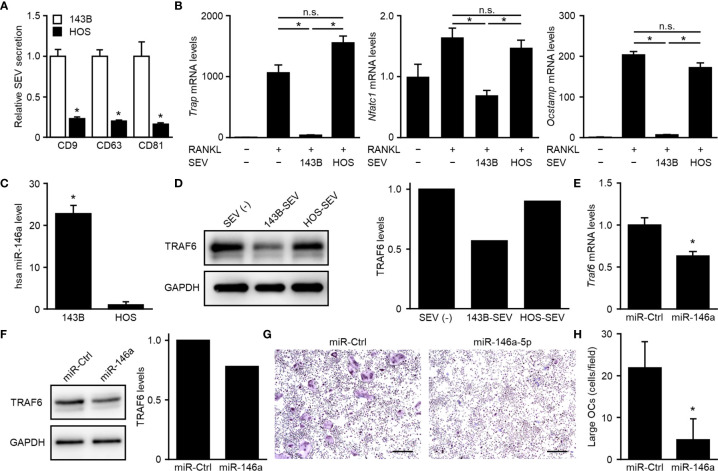
Suppression of osteoclastogenesis dependent on downregulation of TRAF6 by miR-146a-5p. **(A)** CD9-, CD63-, or CD81-positive SEVs secreted from 143B cells or HOS cells were detected in SEV-ELISA. Values represent the mean ± s.d. *P < 0.01 versus 143B cells (Student’s *t*-test). **(B)** OPCs were cultured for 5 days in media with or without each SEVs and with or without RANKL. The relative expression of *Trap*, *Nfatc1*, *Ocstamp* and *Gapdh* genes was detected by real-time PCR. *Trap*, *Nfatc1*, or *Ocstamp* expression was normalized to *Gapdh* expression and is represented as a relative value to that of the RANKL(−), SEV(−) group. Values represent the mean ± s.d. *P < 0.01, n.s., not significant versus the RANKL(+), SEV(−) group or the RANKL(+), 143B-SEV group (Student’s *t*-test with Bonferroni correction). **(C)** Small RNAs were isolated from 143B-SEV or HOS-SEV and subjected to real-time PCR detecting hsa miR-146a-5p and U6. The hsa miR-146a-5p expression was normalized to the U6 expression and is represented as a relative value to that of HOS-SEV. Values represent the mean ± s.d. *P < 0.01 versus HOS-SEV (Student’s *t*-test). **(D)** OPCs were cultured for 2 days in media containing M-CSF with or without each SEVs. The protein of TRAF6 and GAPDH was detected in western blot. The band intensities were normalized to GAPDH. Relative values to the SEV(−) are shown in the bar graphs. **(E, F)** OPCs were transfected with miR-control (miR-Ctrl) or hsa miR-146a-5p mimic and cultured for 2 days in media with M-CSF. The relative expression of *Traf6* and *Gapdh* genes was detected by real-time PCR. *Traf6* expression was normalized to *Gapdh* expression and is represented as a relative value to that of miR-Ctrl **(E)**. The protein of TRAF6 and GAPDH was detected in western blot. The band intensities were normalized to GAPDH. Relative values to the miR-Ctrl are shown in the bar graphs **(F)**. **(G)** OPCs were transfected with miR-Ctrl or hsa miR-146a-5p mimic and cultured for 5 days in media with RANKL. Images of TRAP-staining were taken at ×4 magnification. Scale bars represent 500 µm. **(H)** The area of TRAP-positive cells shown in **(G)** was measured. Numbers of large OCs with ≥ 10,000 μm^2^ per field were calculated. Values represent the mean ± s.d.*P < 0.01 versus miR-Ctrl (Student’s *t*-test).

We next evaluated whether the number of SEVs secreted or the suppressive effect of SEVs correlated with tumor malignancy in human OS. A comparison of two human OS cell lines known to differ in malignancy (20, 21) indicated that highly malignant 143B cells secreted approximately 5-fold higher numbers of SEVs than lowly malignant HOS cells (**Figure 4A**) and that 143B-SEVs suppressed the expression of osteoclastogenesis-related genes more strongly than HOS-SEVs (**Figure 4B**). These results are consistent with the results of tests on mice implanted with TSG101-KO cells. To clarify the mechanism by which 143B-SEVs suppress osteoclastogenesis, we comprehensively analyzed the miRNAs contained in HOS-SEVs and 143B-SEVs by using a micro array analysis. As a result, we identified 16 miRNAs that were > 5 times more abundantly contained in 143B-SEVs than in HOS-SEVs (**Table 1**). Hsa-miR146a-5p is known to target *TRAF6* gene (29), which transduces RANK activation to NF-kB pathway (30, 31). On confirmation by a real time-PCR analysis, it was revealed that hsa-miR146a-5p was 22.8 times more abundant in 143B-SEVs than in HOS-SEVs (**Figure 4C**). Treatment of 143B-SEVs reduced expression of TRAF6 protein compared to that of HOS-SEVs in OPCs (**Figure 4D**). Next, we evaluated the effect of hsa-miR146a-5p on osteoclastogenesis. Transfection of hsa-miR146a-5p mimic reduced the expression of *Traf6* mRNA by 37% compared to that of miR-Ctrl, which caused a decrease in TRAF6 protein (**Figures 4E, F** **)**. Further analysis in TRAP staining also showed that the transfection of miR-146a-5p mimic dramatically inhibited osteoclastogenesis (**Figures 4G, H** **)**. These results indicated that the suppression of TRAF6 expression by miR-146a-5p might be one of the mechanisms by which SEVs from highly malignant OS reduced mature OCs.

**Table 1:** Microarray analysis of miRNA contained in HOS- or 143B-SEVs.

miRNA	Ratio(143B/HOS)	Global normalization	
		HOS-SEV	143B-SEV
hsa-miR-1260a	17.80	154.65	2753.00
hsa-miR-487b-3p	10.12	8.55	86.55
hsa-miR-6720-3p	8.32	1.27	10.60
hsa-miR-146a-5p	7.73	1.48	11.44
hsa-miR-1260b	7.35	1393.98	10245.03
hsa-miR-4758-3p	7.28	80.08	582.65
hsa-miR-4690-3p	7.27	21.44	155.99
hsa-miR-4286	7.11	335.97	2387.88
hsa-miR-6765-3p	6.80	674.17	4581.68
hsa-miR-1261	6.64	1.04	6.93
hsa-miR-7975	6.46	2915.21	18840.32
hsa-miR-4664-5p	6.32	32.89	207.93
hsa-miR-3907	6.14	99.12	608.50
hsa-miR-7977	5.99	3322.24	19905.50
hsa-miR-1273c	5.60	36.73	205.52
hsa-miR-375-5p	5.13	649.45	3331.37

### A Decreased Number of Mature OCs Predicts Poor Prognosis in OS Patients

**Figure 5:**
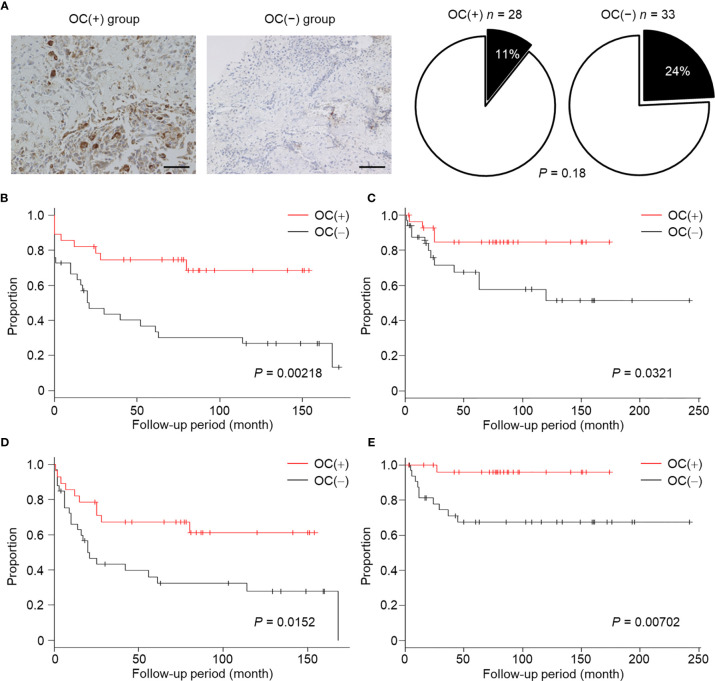
Relationship between decreased number of OCs and poor prognosis in OS patients. **(A)** Biopsy specimens were collected from chemotherapy-naïve OS patients at the time of first diagnosis, and were subjected to IHC staining for cathepsin K. Images were taken at ×20 magnification. Scale bars represent 100 µm. OC(+) group; ≥ 5 OCs/field, OC(−) group; < 5 OCs/field. Patients with distant metastasis at first visit are shown as a black part. **(B–E)** Metastasis-free survival **(B)**, recurrence-free survival **(C)**, progression-free survival **(D)**, and overall survival **(E)** in the OC(+) group or OC(−) group were followed up. The proportions and times were plotted as Kaplan–Meier curves. *P* values were calculated between the OC(+) group and OC(−) group (log-rank test).

**Figure 6:**
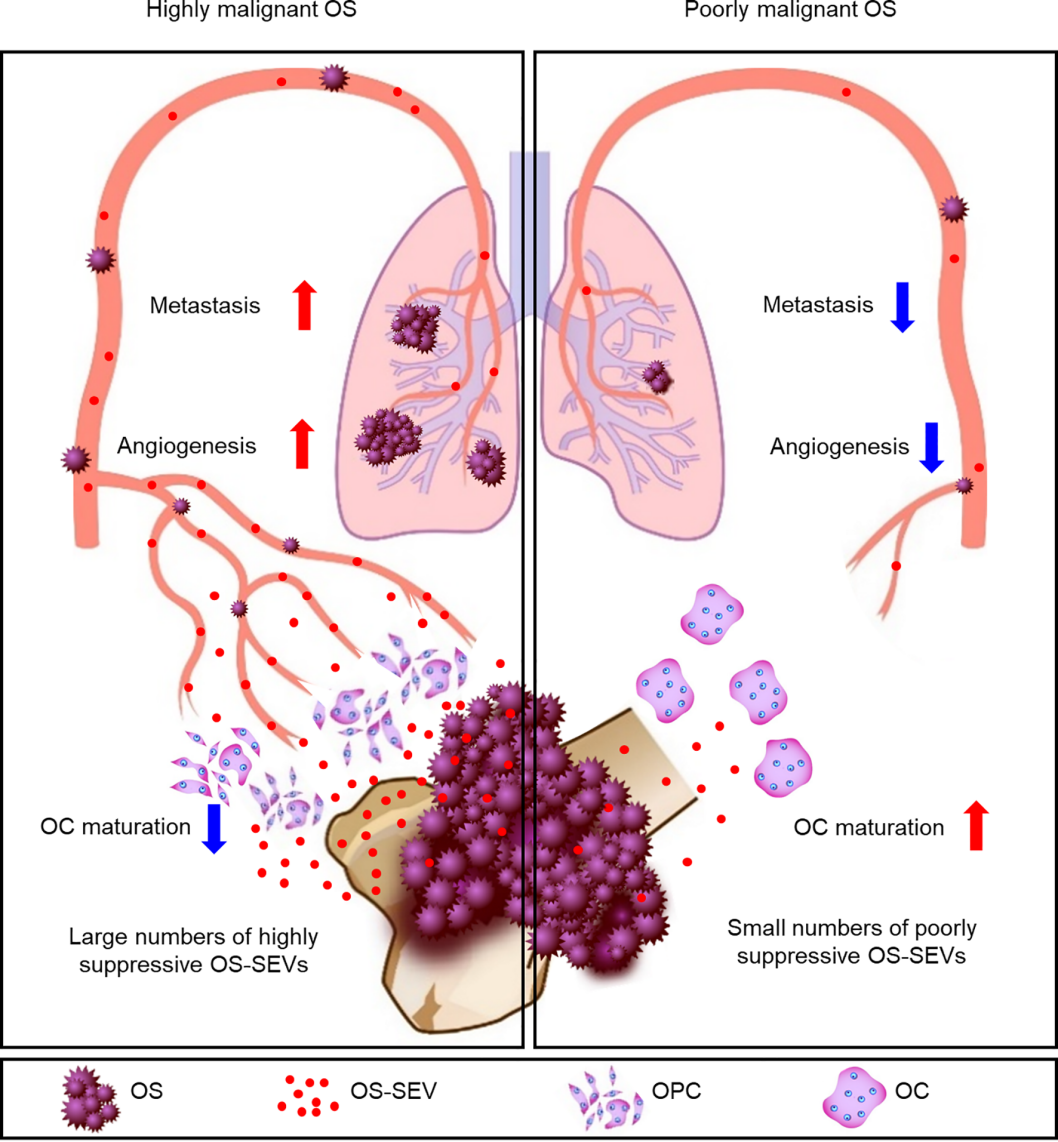
Schematic representation of the key findings. In patients with highly malignant OS, a large number of highly suppressive SEVs inhibit osteoclastogenesis and enhance angiogenesis and metastasis. In patients with poorly malignant OS, a small number of poorly suppressive SEVs rarely inhibits osteoclastogenesis, resulting in a reduced incidence of metastases.

Further, we hypothesized that highly malignant OS might secrete a large number of highly suppressive SEVs, causing a decreased number of mature OCs in patients. To determine the relationship between OS malignancy and OC numbers, we investigated the number of OCs in chemotherapy-naïve patient specimens and followed up their clinical outcomes based on 61 patients with OS. Based on IHC for cathepsin K, patients were categorized as follows: those with < 5 mature OCs per field of view as OC(−) patients (*n* = 33), and patients with ≥ 5 mature OCs per field of view as OC(+) patients (*n* = 28) (**Figure 5A**). All patient characteristics were not significantly different between the two groups (**Table 2**). According to the results of multimodal radiological examinations, 24% of OC(−) patients and 11% of OC(+) patients had distant metastasis, indicating no significant difference in metastasis rates between the two groups at first visit (**Figure 5A**). As a result of the follow-up study, we found that metastasis-free survival, recurrence-free survival, progression-free survival, and overall survival were significantly prolonged in OC(+) patients (**Figures 5B–E**). Similarly, metastasis and recurrence occurred at a significantly higher rate in OC(−) patients than in OC(+) patients (73% vs 29%, and 36% vs 14%, respectively), and mortality from the disease was also higher in OC(−) patients (30%) than in OC(+) patients (4%) (**Table 2**). These results indicated that the number of mature OCs around OS lesions were related to OS malignancy. Finally, we evaluated the accuracy of the test to predict metastasis (**Table 3**). The analysis of diagnostic accuracy showed that a decrease in mature OCs based on cathepsin K IHC was a valuable predictive indicator for metastasis associated with OS, with a sensitivity of 75% and a specificity of 69% (**Table 4**). The diagnostic accuracy of this method was 72%, indicating that it is moderately appropriate for the screening of high-risk patients for metastasis. In conclusion, we showed that many highly suppressive SEVs secreted from highly malignant OS suppress osteoclastogenesis and enhance distant metastasis in mice and humans (**Figure 6**). Additionally, examining the number of mature OCs around an OS lesion should be helpful to predict prognosis.

**Table 2:** Patients’ characteristics.

	OC(+) group	OC(−) group	*P* value
**Case number (*n*)**	28	33	–
**Age (year)**	29±23	32±23	0.58
**Sex (male/female)**	17/11	18/15	0.60
**Follow-up periods (month)**	84±44	86±70	0.91
**Location**			
Appendicular skeleton	23	24	0.39
Axial skeleton	5	6	1.00
Others	0	3	0.10
**Tumor size** **(greatest dimension, mm)**	87±34	108±91	0.13
**Type**			
Osteoblastic	9	13	0.59
Chondroblastic	4	8	0.23
Fibroblastic	2	3	0.78
Small cell	0	1	0.31
Giant cell rich	1	0	0.31
Telangiectatic	2	0	0.11
Unknown	10	8	0.33
**Stage**			
IIA	12	8	0.13
IIB	13	13	0.56
III	0	2	0.19
IVA	3	8	0.18
Unknown	0	2	0.19
**Treatment**			
Surgery	25	29	0.87
Neoadjuvant chemotherapy	24	22	0.08
Adjuvant chemotherapy	27	27	0.08
Radiation	2	5	0.34
Others	0	2	0.19
**Efficacy of chemotherapy** **(Rosen & Huvos)**			
I – II	8	14	0.04
III – IV	15	6	0.02
Unknown	1	2	0.91
**Outcomes**			
Metastasis during follow-up periods	8	24	<0.01
Recurrence during follow-up periods	4	12	0.05
Disease-free at final follow-up	24	29	0.02
Dead of disease	1	10	<0.01

**Table 3:** Cross-classification of OS patients by IHC of cathepsin K and metastasis.

	Metastasis during follow-up periods		Total
	Positive	Negative	
**IHC of cathepsin K**			
** OC(+)** ** OC(**−**)**	8 24	20 9	28 33
**Total**	32	29	61

**Table 4:** Diagnostic accuracy measures.

	Estimate	95% Confidence interval	
		Lower limit	Upper limit
**Positive ratio**	0.541	0.408	0.669
**True positive ratio**	0.525	0.393	0.654
**Sensitivity**	0.750	0.566	0.885
**Specificity**	0.690	0.492	0.847
**Positive predictive value**	0.727	0.545	0.867
**Negative predictive value**	0.714	0.513	0.868
**Diagnostic accuracy**	0.721	0.592	0.829
**Positive likelihood ratio**	2.417	1.355	4.309
**Negative likelihood ratio**	0.362	0.190	0.693

## Discussion

The OS microenvironment is composed of different stromal cell types and contributes to proliferation, angiogenesis, and metastasis (6, 15). Although many effects of tumor-derived SEVs on development of the tumor microenvironment have been reported in various tumors, little is known about the effect of SEVs in OS. We analyzed phenotypes of SEV-depleted OS cells in a murine OS model, and revealed that OPCs are active receivers of OS-SEVs in the microenvironment, causing a decrease in the number of mature OCs and increase in undifferentiated OPCs. OPCs can potentially secrete platelet-derived growth factor-BB (PDGF-BB), which induces the angiogenesis of CD31-positive vessels (32, 33). Thus, the increased angiogenesis seen in mice implanted with LM8-WT cells might be attributed to PDGF-BB secretion from OPCs induced by OS-SEVs.

Little is known about the function of OCs in the OS microenvironment. However, Endo-Munoz et al. have reported on this (11). Depletion of OCs *via* the injection of ZA dramatically enhanced OS lung metastasis in mice. Furthermore, the coculture of OS cells and OPCs inhibited the generation of mature OCs *in vitro*, but the authors did not identify any inhibitors secreted from OSs. In our study, we identified OS-SEVs as a promising candidate for OS-derived factors that suppress osteoclastogenesis. Endo-Munoz et al. also reported that ZA treatment increases the incidence of lung metastases in some OS cell lines (e.g. 143B cells) but not in others (e.g. HOS cells) (11). In our study, 143B cells secreted more OS-SEVs than did HOS cells, and 143B-SEVs had an increased ability to suppress osteoclastogenesis than did HOS-SEVs. It is possible to hypothesize that ZA may promote OS progression, not in all patients, but in patients with OSs that secrete many highly malignant OS-SEVs. This hypothesis might help provide an answer to the unexpected results observed in the clinical trials on ZA.

To identify a molecule suppressing osteoclastogenesis, we performed the microarray analysis of miRNAs. Although several miRNAs, including miR-7b, miR-124, and miR-223, are known to suppress osteoclastogenesis, they were not detected in our microarray analysis (34–36). We identified that miR-146a-5p contained in OS-SEVs has the potential to inhibit osteoclastogenesis by suppressing *TRAF6* mRNA expression. The significance of miR-146a-5p in the proliferation of OS (37) and the involvement of miR-146a-5p in angiogenesis (38, 39) have been reported. We showed that miR-146a-5p affects not only tumor cells and vascular endothelial cells, but also OPCs constituting the tumor microenvironment. Because it is difficult to isolate OS-EVs from tumor microenvironment, we could not show the relationship between miR-146a-5p and OC number in OS patients but showed that reduction in OC numbers was associated with accelerated metastasis and worse prognosis in OS patients. It is surprising that the number of OCs in biopsy specimens collected at the time of first diagnosis could accurately predict future metastasis and overall survival. OCs in the tumor microenvironment are the first cells affected by OS-EVs, hence decrease in OCs might occur earlier than angiogenesis and metastasis. It might enable early diagnosis to predict metastasis and overall survival in the OS patients. Further studies on miR-146a-5p contained in OS-EVs in patients will contribute to the development of more accurate prognosis as well as new therapies.

Endo-Munoz’s group designed a similar clinical study (*n* = 22), which indicated a correlation between *TRAP* mRNA expression and time to metastasis (*P* = 0.0166) but not between *TRAP* mRNA expression and overall survival (11). In this study, we focused stringently on the numbers of mature OCs by measuring the number of cathepsin K-positive cells in the biopsy specimens. Considering that OS-SEVs suppress osteoclastogenesis, measuring the size of mature OCs might be more diagnostic than measuring *TRAP* mRNA expression. We developed a simple diagnostic method using the conventional biopsy, which shows good accuracy for the prediction of metastasis. This will help provide optimal care to patients with a high risk of malignancy. Additionally, targeting OS-SEVs might become a promising therapeutic strategy to prevent progression or metastasis in OS. As such, further study on OS-SEVs or OCs in the tumor microenvironment might improve the prediction of tumor progression or aid in the development of novel treatments.

## Data Availability Statement

The datasets presented in this study can be found in online repositories. The names of the repository/repositories and accession number(s) can be found in the article/**Supplementary Material**.

## Ethics Statement

The studies involving human participants were reviewed and approved by Ethical Committee of Kanazawa University Hospital. Written informed consent to participate in this study was provided by the participants’ legal guardian/next of kin. The animal study was reviewed and approved by Institutional Animal Care of Kanazawa University.

## Author Contributions

HA, TY, and RH designed the research. YA, HA, TY, and TN performed the research. YA, HA, ST, and TN analyzed the pathological specimens. KIs directed the osteoclastogenesis protocol. YA, HA, TY, and RH analyzed the data and wrote the paper. NY, KH, AT, SM, HM, KIg, and HT supervised the research and reviewed the original paper. All authors contributed to the article and approved the submitted version.

## Funding

This work was supported by Core Research for Evolutional Science and Technology (CREST) from Japan Science and Technology Agency (JST) (No. JPMJCR18H4 to RH), Grants-in-Aid for Scientific Research (KAKENHI) from the Ministry of Education, Culture, Sports, Science and Technology (MEXT) (No. 70793834 to HA, No. 10800625 to YA, and No. 19H03433 to RH), Children’s Cancer Association of Japan (to HA), and the World Premier International Research Center Initiative (WPI), MEXT, Japan.

## Conflict of Interest

The authors declare that the research was conducted in the absence of any commercial or financial relationships that could be construed as a potential conflict of interest.
