# Supplementary material for: Osteosarcoma-Derived Small Extracellular Vesicles Enhance Tumor Metastasis and Suppress Osteoclastogenesis by miR-146a-5p
Source: Front Oncol. 2021 May 4;11:667109. doi: 10.3389/fonc.2021.667109 (PMC8130824; doi:10.3389/fonc.2021.667109)
Supplement: Supplementary file 1 [file DataSheet_1.pdf]

Supplementary Figures and Tables

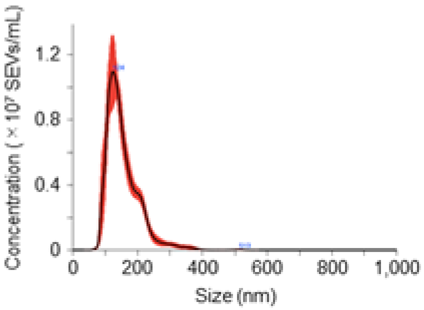

Supplementary Figure 1. Size distribution of LM8-SEVs. SEVs were isolated from cultured supernatant of LM8 cells by ultra-centrifugation. After washing with PBS(-), LM8-SEVs were re-suspended in PBS(-). Size distribution of the LM8-SEVs was analysed three times in nanoparticle tracking analysis. Black line and red zone represent mean and s.d. respectively.

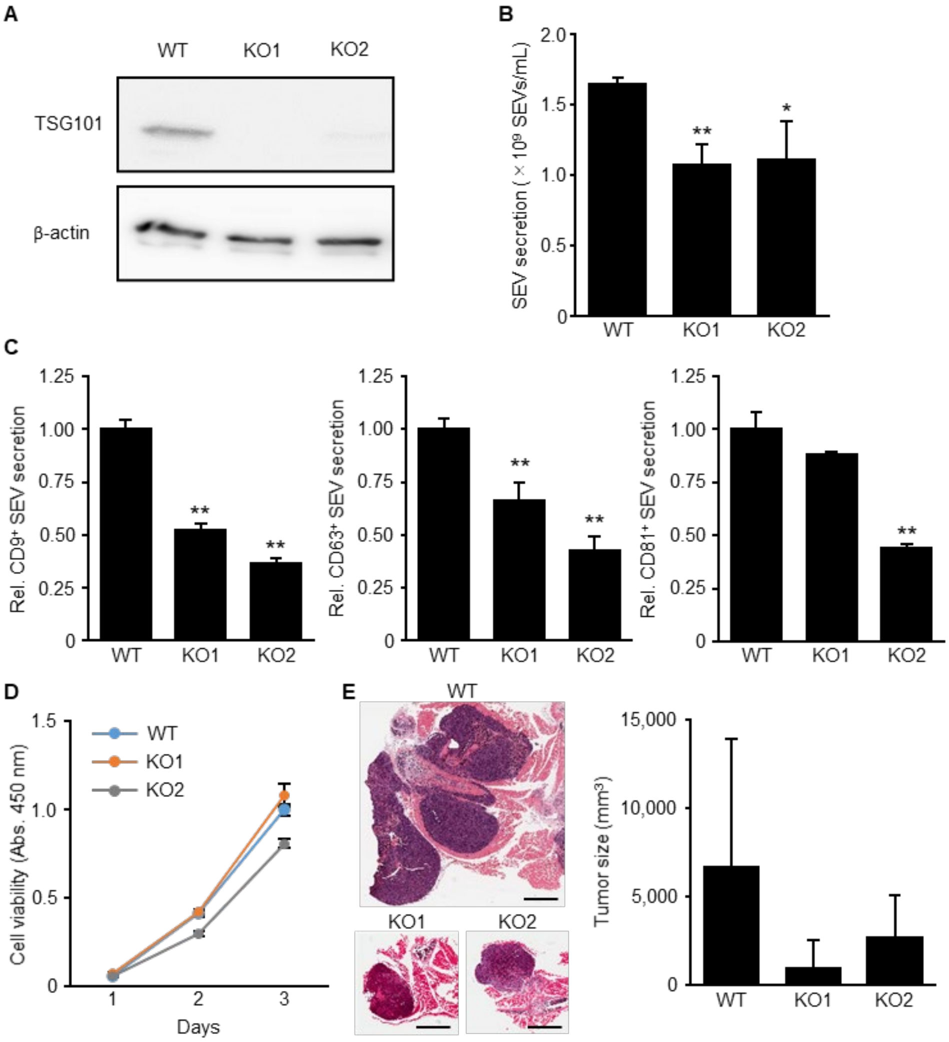

Supplementary Figure 2. Features of LM8 TSG101-KO cells *in vitro* and *in vivo*. (**A**) LM8 WT, TSG101-KO1, or TSG101-KO2 cells were lysed in RIPA buffer. The lysates were subjected to western blot analysis using monoclonal antibodies against TSG101 (clones 4A10, Sigma–Aldrich), anti-β-actin (AC-15, Sigma–Aldrich). (**B**,**C**) LM8 WT, TSG101-KO1, or TSG101-KO2 cells were seeded at 1×10^5 cells/mL. After a 24-h culture, the cultured media were centrifuged to remove cells, debris, and large EVs. SEVs secreted into the cultured medium were quantified by a nanoparticle tracking system (**B**) or SEV-ELISA using anti-mouse CD9, anti-mouse CD63, or anti-mouse/rat CD81 antibody for detection ($n = 3$) (**C**). Values represent mean ± s.d. *$p < 0{\cdot}05$, **$p < 0{\cdot}01$ versus WT (Student's *t*-test). (**D**) LM8 WT, TSG101-KO1, or TSG101-KO2 cells were seeded at 2×10^3 cells/well. After culture for indicated days, cellular growth was determined by WST-8 assay ($n = 3$). Values represent mean ± s.d. (**E**) For tumor-forming assay, 1×10^5 cells of LM8 WT, TSG101-KO1, or TSG101-KO2 cells were implanted in distal femurs of C3H/He mice. Four weeks after implantation, the femurs were subjected to H&E staining, and the tumor size was measured ($n = 7$, WT and KO1; $n = 8$, KO2). Values represent mean ± s.d. Scale bars represent 3 mm.

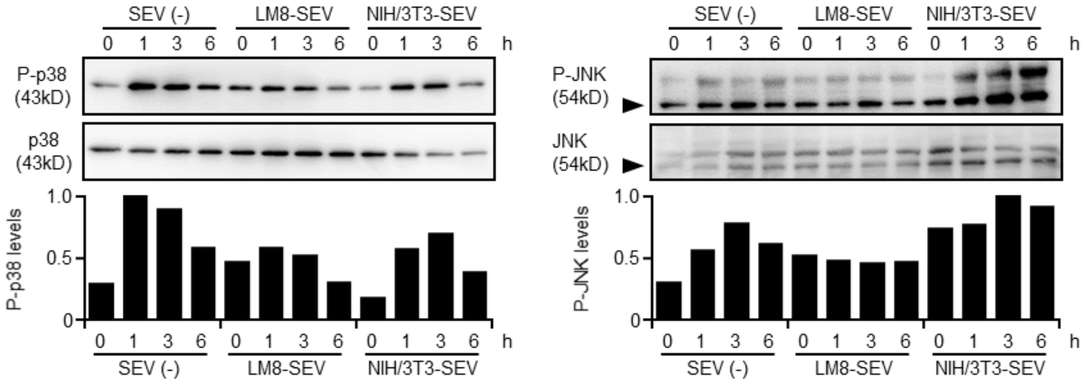

Supplementary Figure 3. Suppression of MAPK pathway by LM8-SEVs. OPCs were pre-cultured in media without SEVs or with LM8-SEVs or NIH/3T3-SEVs for 12 h. After RANKL stimulation for the indicated time, OPCs were harvested and subjected to western blot. Detection of JNK, phospho-JNK, p38, phospho-p38 and GAPDH proteins was performed using polyclonal antibodies against SAPK/JNK, phospho-SAPK/JNK (Thr183/Tyr185), p38, and phospho-p38 (Thr180/Tyr182) (all from Cell Signaling Technology). Band intensities of phospho-p38, or phospho-JNK were measured and normalized to GAPDH in Fig. 3**C**. Relative values to the highest sample are shown in the bar graphs.

Supplementary Table 1. Sequence of primers for real-time PCR

Gene	Forward primer	Reverse primer
Trap (Acp5)	GCGACCATTGTTAGCCACATACG	CGTTGATGTCGCACAGAGGGAT
Atp6v0d2	AGCCCCTGAGCACATTTCT	CAGTGGGTGACACTTGGCTA
c-fos	ATGGGCTCTCCTGTCAACAC	CCACGGAGGAGACCAGAGT
Dcstamp	TGTGCTTGTGGAGGAACCTA	AGACTCCCAAATGCTGGATG
Nfatc1	AGTCTCTTTCCCCGACATCA	TCATAGTGAGCCCTGTGGTG
Ocstamp	GCCTCCAGAACACAGCTTTC	AACAACTGCCTTGCAGATCC
Gapdh	GTGTTTCCTCGTCCCGTAGA	AATCTCCACTTTGCCACTGC
hsa-miR-146a	TGAGAACTGAATTCCATGGGTTA	Included in the kit
U6	Included in the kit	Included in the kit
